# Multiple perspectives on symptom interpretation in primary care research

**DOI:** 10.1186/1471-2296-14-167

**Published:** 2013-11-04

**Authors:** Marianne Rosendal, Dorte Ejg Jarbøl, Anette Fischer Pedersen, Rikke Sand Andersen

**Affiliations:** 1 Research Unit for General Practice, Department of Public Health, Aarhus University, Bartholins Alle 2, DK-8000 Aarhus C, Denmark; 2 Research Unit of General Practice, Institute of Public Health, University of Southern Denmark, J.B. Winsløws Vej 9 A, DK-5000 Odense, Denmark

**Keywords:** Symptom research, Signs and symptoms, Symptom assessment, Interdisciplinary studies, General practice, Medicine, Psychology, Anthropology

## Abstract

**Background:**

Assessment and management of symptoms is a main task in primary care. Symptoms may be defined as 'any subjective evidence of a health problem as perceived by the patient’. In other words, symptoms do not appear as such; symptoms are rather the result of an interpretation process. We aim to discuss different perspectives on symptom interpretation as presented in the disciplines of biomedicine, psychology and anthropology and the possible implications for our understanding of research on symptoms in relation to prevalence and diagnosis in the general population and in primary care.

**Discussion:**

Symptom experiences are embedded in a complex interplay between biological, psychological and cultural factors. From a biomedical perspective, symptoms are seen as possible indicators of disease and are characterized by parameters related to seriousness (e.g. appearance, severity, impact and temporal aspects). However, such symptom characteristics are rarely unambiguous, but merely indicate disease probability. In addition, the GP’s interpretation of presenting symptoms will also be influenced by other factors. From a psychological perspective, factors affecting interpretation are in focus (e.g. internal frame of reference, attention to sensations, illness perception and susceptibility to suggestion). These individual factors cannot stand alone either, but are influenced by the surroundings. Anthropological research suggests that personal experiences and culture form a continuous feedback relationship which influence when and how sensations are understood as symptoms of disease and acted upon.

**Summary:**

The different approaches to symptom interpretation imply that we need to be cautious and conscious when interpreting survey findings that are based on symptom prevalence in the general population or in primary care. These findings will reflect a variety of interpretations of sensations, which are not equivalent to expressions of underlying disease. Furthermore, if diagnosis of disease is based exclusively on the presence of specific symptom characteristics, we may risk reinforcing a dualistic approach, including medicalisation of normal phenomena and devaluation of medically unexplained symptoms. Future research in primary care could gain from exploring symptoms as a generic phenomenon and raised awareness of symptom complexity.

## Background

**Figure 1:**
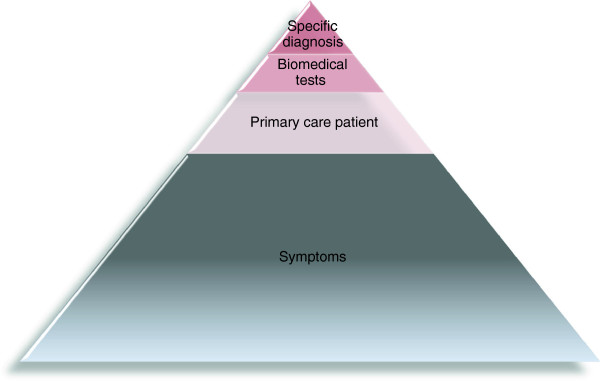
The symptom iceberg.

People present with symptoms in primary care every day, and there is a need for research in symptoms as a generic phenomenon [1]. Studies have so far mainly focused on symptoms as manifestations of well-defined disease, but during the last three decades we have witnessed an increase in population-based studies exploring symptom prevalence in different community settings [2-6]. The experience of symptoms is common, but symptoms are often ignored by the individual or cared for in a private setting. Only few cases are presented to the general practitioner (GP); a phenomenon known as the ’symptom iceberg’ [2] (Figure 1). For example, a recent British study of 1000 adults confirmed that 9 of 10 adults had experienced some sort of symptom or ailment within the last two weeks. Only 10% of these saw a doctor, and 46% did not take any action [3]. A recent Danish study found that even symptoms of potentially severe disease are common: 15% reported to have experienced at least one of four cancer alarm symptoms (lump in the breast, cough for more than 6 weeks, blood in the urine, rectal bleeding) during one year [7]. Numerous studies have tried to estimate the prevalence of different parts of the symptom iceberg, but - as will appear from our discussion - such estimates will also depend on the perspective taken.

**Figure 2:**
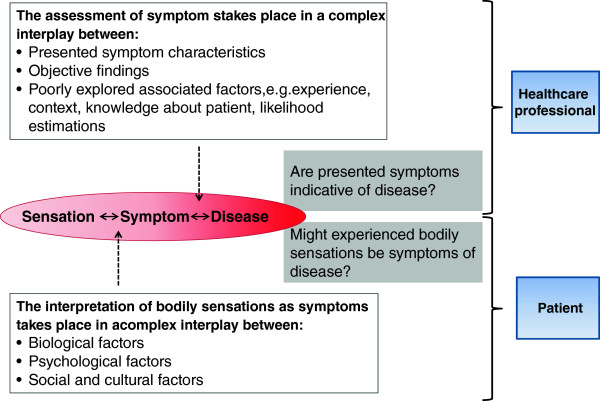
Symptom interpretation and behaviour in the medical context.

The WONCA Dictionary of General/Family Practice defines symptoms as 'any subjective evidence of a health problem as perceived by the patient’ [8]. This definition implies that symptoms do not appear as such; rather symptoms are the result of an interpretation process ('any subjective evidence’), where sensations are transformed into signs of ill-health. In order to understand the implications of the symptom iceberg, we must further address how people interpret and respond to bodily sensations such as symptoms. In addition, we must also identify factors determining when or how a sensation is experienced a symptom and acted upon (see Figure 2).

Why is this of importance to general practice? The interface between sensations, symptoms and potential disease is interpreted differently, depending on your focus and approach to the symptom iceberg (i.e. whether you belong to the general population, are an individual seeking health care or a patient referred to secondary care). As general practice is at the border between the population and the health care system, primary care research will necessarily touch upon aspects of all of these populations and the characterisation of symptoms will depend on who is making this interpretation, in which context and from which perspective.

### Aim

We aim to discuss different perspectives on symptom interpretation as presented in the disciplines of biomedicine, psychology and anthropology. In particular, we will focus on how different disciplines may bring forth new insight about research on symptoms of importance to primary care. This may provide a first step towards a more systematic approach to future symptom research.

## Discussion

In the following three sections, we will describe the perspectives taken on the interpretation of symptoms in the disciplines of biomedicine, psychology and anthropology. Each field tends to have its own distinctive scientific approach and terminology. When discussing symptoms from three distinctive perspectives, we may risk to enforce traditional dualistic thinking between mind and body (and context). The clear distinctions brought forward in the discussion presented here are primarily established for the sake of the argument. Likewise, we have chosen to discuss only bodily symptoms and not include mental symptoms.

### The biomedical perspective on symptom interpretation

Early diagnosis and prompt treatment are considered the key to a better prognosis. Hence, symptom interpretation as presented in the discipline of biomedicine is influenced by a desire to predict risks of negative health effects and is based upon certain characteristics related to seriousness. From the biomedical perspective, seriousness is mostly thought of in terms of objective pathology rather than subjective experience. Based on this, biomedicine focus on certain symptom characteristics presented below.

#### ***Symptom nature and appearance***

We distinguish between subjective health complaints and signs; the latter being objectively verifiable (e.g. blood in the urine or jaundice). Signs are seen as reliable markers of disease, whereas symptoms often refer to subjective complaints. However, even signs may be located in a continuum between disease biomarker and medically unexplained symptoms (e.g. various degrees of cough and oedema).

Symptoms may appear singly (e.g. headache) or in clusters of symptoms (e.g. rheumatoid arthritis). Although a high symptom burden or a specific symptom pattern may indicate higher risk of persistence and disorder [9,10], many diseases lack differentiated symptoms or signs [11]. Even well described symptom patterns for severe diseases may be similar to, for example, functional somatic syndromes (e.g. some of the alarm symptoms of colon cancer are included in the diagnostic criteria for irritable bowel syndrome) [12].

In biomedicine, objective signs and specific symptom clusters are seen as reliable indicators of disease and consequently, they are often given higher priority than the subjective symptom experience (as described in for example the clinical assessment of cough [13]).

#### ***Symptom severity and impact on daily life***

People are more likely to seek health care if symptoms are perceived as severe or incapacitating [2], and the clinicians’ diagnostic and treatment decisions also consider the patient-perceived severity [14]. However, symptom severity can rarely be measured objectively, but relies heavily on the subjective assessment; as stated above, severity and impact on daily life may thus be given lower priority from a biomedical perspective. Nevertheless, the literature emphasises symptom severity as a phenomenon that should be seen in a broader multi-component construct involving integration of patient-reported severity ratings in combination with other clinical measures, such as daily functional status or concurrent psychosocial features [14].

#### ***Temporal aspects***

Symptom onset, duration and possible frequency and fluctuations over time form part of the symptom pattern. However, the correlation between time and disease is not unambiguous. Yet, GPs tend to react with biomedical tests if symptoms persist or progress [15]. Many guidelines tend to encourage the interpretation of seriousness simply based on duration, as they instruct GPs to pursue symptoms and signs lasting for more than a predefined interval [16]. Furthermore, several diagnostic criteria include duration as a parameter.

In conclusion, symptom characteristics are hardly ever unambiguous, and mostly the characteristics merely indicate a given probability of disease. For example, much focus has been placed on symptoms that are indicative of cancer. However, the positive predictive values of most cancer alarm symptoms are low, both in the general population and in primary care and the evidence base for using alarm symptoms to identify cancer is weak [17-19].

When GPs face patients presenting with symptoms, they base their evaluation and subsequent actions on symptom characteristics and predictive values using a biomedical approach (Figure 2). This may seem straightforward, but as symptom characteristics are often ambiguous, the interpretation will also be affected by individual factors, culture and context. This implies that the GP’s collection and analysis of information during the consultation is affected by factors such as own knowledge, previous experience and general knowledge of the patient [20]. The possible impact of these and other factors (some of which will be described below) on symptom interpretation in primary care is poorly explored.

### The psychological perspective on symptom interpretation

**Figure 3:**
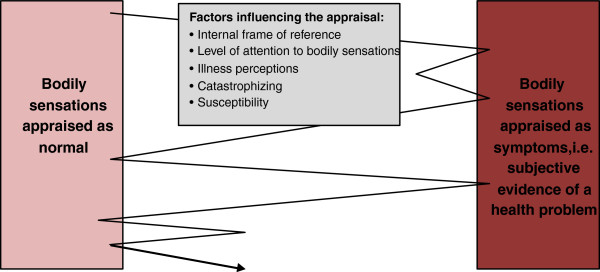
Examples of psychological factors influencing the appraisal of bodily sensations.

Bodily sensations alert us against potentially damaging stimuli. Results of laboratory studies have revealed fairly uniform pain thresholds. Hence, a one-to-one relation between tissue injury and pain experience has for many years dominated the scientific approach in pain research involving physical stimuli [21]. However, researchers have discovered that this purely sensory approach to bodily sensations cannot stand alone since it does not capture the great variability in pain intensity reported by different individuals; sometimes variations occur even for the same person at different time points. Numerous psychological factors have been suggested to moderate the experience and interpretation of bodily sensations. Some of the scientifically most investigated factors are described below (please refer to Figure 3 for an illustration).

#### ***An internal frame of reference***

Our interpretation of bodily sensations as either 'normal’ or 'threatening’ is moderated by an internal frame of reference based on our previous bodily experiences. For instance, hemiplegia may be considered normal to a person suffering from familial hemiplegic migraine, but would cause high levels of worries if experienced by a healthy person. A painful chronic condition does not imply that the suffering person habituate to high levels of pain, as one might expect. On the contrary, patients with rheumatoid arthritis have been shown to exhibit an enhanced reactivity to pain (general hyperalgesia) compared to healthy individuals [22]. Thus, an internal frame of reference developed on the basis of a chronic condition seems to lower the pain threshold, but also to broaden the perception of 'normality’.

#### ***Attention to bodily sensations***

Different people pay different attention to bodily sensations. In order to describe individual differences, various concepts ─ such as somatosensory amplification ─ must be considered [23]. Somatosensory amplification is the tendency to experience a bodily sensation as intense, noxious and disturbing and to perceive every bodily sensation as abnormal, pathological and a symptom of disease [23]. Somatosensory amplification, assessed by the Somatosensory Amplification Scale (SSAS), has been positively associated with high levels of health worries and hypochondriacal behaviours [24]. However, one study did not confirm an association between scores on the SSAS and heartbeat detection ability or sensitivity to stimuli originating from the inside of the body [25]. This particular finding raises the question whether the increased levels of health worries observed in persons with high scores on the SSAS are triggered by stimuli from the body or rather reflect a personality trait operating independently of actual physical stimulation.

#### ***Illness perceptions***

When faced with a health threat such as an unexplained symptom or a diagnosis, individuals will develop cognitive models of this threat, and these mental representations will guide the individual’s response to the health threat [26]. The cognitive models – or 'illness perceptions’ – are based on e.g. the individual’s own medical knowledge, internal frame of reference, and family members’ or friends’ experiences with similar symptoms. Illness perceptions are often concentrated on five interrelated concepts: causal beliefs, timeline beliefs, beliefs about control, consequences and identity of the symptom/illness [27]. In patients diagnosed with various diseases, negative illness perceptions have been associated with a number of clinical outcomes (such as slower recovery, future disability, increased healthcare use, and lack of reassurance) when physical examinations reveal no pathology [27]. Most of the research conducted within the field of illness perceptions has focused on patients who have already been diagnosed with a disorder, and little is known about the role of symptoms.

Catastrophizing is an automatic cognitive style characterized by 'the tendency to magnify the threat value of pain stimulus and to feel helpless in the context of pain, and by a relative inability to inhibit pain-related thoughts in anticipation of, during or following a painful encounter’ [21]. The concept is often thought to be a component of illness perceptions such as controllability and consequences [28]. The tendency to catastrophize when exposed to pain has been shown to be associated with altered CNS pain processing in the form of reduced endogenous pain inhibition, but the causal nature of this relationship remains to be determined. In their interpretation of bodily sensations, individuals also include external sources of information. Occasionally, media stories cause a great number of persons to report symptoms without any evidence of pathology. Examples of this phenomenon called 'symptoms by suggestion’ are: the wind turbine syndrome [29] and the sick building syndrome [30].

In conclusion, the interpretation of bodily sensations is affected by a number of psychological factors such as internal frame of reference, level of attention to bodily sensations and illness perceptions, which are all influenced by e.g. environmental factors. These factors have been described as separate entities above, but will often overlap and influence each other. The scientific approach to a clarification of the role of psychological factors in the interpretation of bodily sensations is often based on a simple stimulus-response model, e.g. how does a psychological factor moderate the response (i.e. the symptom interpretation) when exposed to a stimulus (i.e. the bodily sensation)? However, growing evidence suggests that the causal relationships are more complex than previously thought, and the exposure to bodily sensations is under heavy influence of psychological and other factors [27,31].

### Anthropological perspectives on symptoms

Symptom experiences have been subject to anthropological analysis in different ways, ranging from studies of personal illness narratives [32,33] to macro-level analysis of the development and ramifications of particular illness discourses [34-36] and the cultural and political sources of distress and suffering [37-39], in both homogeneous and hyperdiverse social and cultural settings. In the following brief overview, two main points from the literature will be brought forward: Firstly, symptoms are not conceptualised as objective clinical entities, rather they evolve during the interpretation process in particular cultural settings. Secondly, changes in biomedical thinking have been found influential for providing legitimate categories through which bodily sensations are experienced and presented as symptoms.

#### ***Medical categorization***

Rather than making normative evaluations of the nature of symptoms reported by people (as subjective versus objective signs of disease), anthropologists have traditionally been interested in exploring the inter-relatedness between peoples’ subjective symptom experiences and the biomedical field as a cultural system [35,36,40]. This approach has raised awareness about the role of doctors and biomedical thinking as co-producers of the cultural categories that frame peoples’ bodily experiences and expressions. Bodily experiences do not take place, nor are they expressed in a vacuum; conceptual categories shape our experience and influence our interpretation of bodily experiences. In the Western world, biomedical thinking is a key actor in defining the categories through which we experience and express our bodies [36,38]. Research has illustrated how novel diagnostic categories may even open up new spaces for the articulation of bodily experiences or sensations. Examples include the introduction of menopause as a deficiency disease in the 20^th^ century [36] and PTSD as a diagnostic category embracing the Vietnam veterans in the 1960s and 70s [35]. Others have illustrated how new biomedical knowledge on the immune system [40] or cancer [41] can develop into disease metaphors that tend to shape our views on health and illness. Some have suggested that 'symptom pools’ could be seen as a range of symptom categories that are available to legitimately designate bodily changes or suffering at any given time in any given culture [39]. This is not to argue that people uncritically adopt biomedical categories when experiencing potential illness, but the examples presented above illustrate how the meaning of bodily experiences may be redirected or changed as new biomedical categories emerge. Moreover, these examples remind us that the transformation of bodily sensations into symptoms requires culturally acceptable categories, which may differ geographically and historically.

#### ***The clinical setting***

The issue of legitimacy is also evident in the way people may engage with doctors in the clinical setting. Studies have shown that the particular clinical framing of symptom presentations tend to influence how people present their illness complaints [32,38]. For instance, Kleinman’s studies in Taiwan and Boston illustrate how people may present the same illness complaints in different phrases or wordings, depending on whether they see a biomedical doctor or a CAM healer [38]. Similar findings have been produced in a Danish study [32]. Of equal importance, a large body of literature has documented how cultural differences between patients and doctors may result in different expectations to the encounter, influence the therapeutic alliance, and how illness complaints are presented and validated [42].

#### ***Gender differences***

Other social and cultural elements contribute to shaping our symptom experiences. A vast amount of studies have for example illustrated how gendered roles, and in particular the regulation of the female body, frame bodily experiences [33,36,43,44]. Some argue that health in many western societies has gained symbolic importance influencing cultural values and providing a means of negotiating gendered identities in which masculinity is partly constructed in opposition to the 'healthy beliefs and behaviours’ of women [33,45]. Others demonstrate how women in Western societies are generally expected to be capable of surveilling the health of their own bodies, but also the bodies of their families (the embodiment of obligation). An expectation which both establishes and legitimizes a high degree of bodily awareness, and which influence the way that bodily changes are perceived [39]. Similarly, in a study on pain and gender, Bendelow and Williams argue that in some Western settings women may naturalize pain experiences because they are endowed with 'culturally superior’ pain endurance. Through motherhood and their general role as emotion managers, women are socialised to cope with bodily experiences in a different way than their male counterparts. Therefore, women are more prone to view pain experiences as natural bodily processes that are not necessarily symptoms of underlying disease [33].

#### ***Social relations and sanctioning***

The interpretation of bodily sensations also takes place in a process of social interaction. Some studies suggest that illness has social ramifications, and therefore bodily sensations need to be sanctioned as symptoms in the social arena [46,47]. Having symptoms implies being potentially ill; sometimes this involves release from social obligations and implies the need for care. The experience of illness signs must be agreed by others in order to warrant both these privileges [47]. Others have argued that social interaction is of importance as it precedes meaning-making. For example, Kleinman illustrates that family members not only respond to bodily changes when these appear, but also play an important part in shaping the way that the body is experienced and understood [38].

In summary, anthropological research suggests that bodily experiences and culture should be considered as a continuous feedback relationship in which a specific historical, political or social context contributes to different expectations that influence when and if bodily sensations are seen as symptoms of illness.

### Cross-disciplinary discussion

Symptoms are not merely objective phenomena. Rather, as exemplified in the above, they are multidimensional constructs in which different social and cultural settings as well as different psychological processes may cause bodily sensations to manifest as symptoms or amplify. Although we may describe some typical biomedical characteristics of symptoms, we should be aware of this complexity since it has implications both for research and clinical work in relation to primary care. By exploring the symptom iceberg from bottom to top, we will discuss some of these implications for the interpretation of population studies, clinical practice and diagnostic classification in the health care system (summarized in Table 1).

**Table 1:** Interpretation of symptoms: a summary

	
**The interpretation of symptoms within individual research disciplines**	
Biomedical perspective	• Focus is on interpretation of signs and symptoms as indicators of disease according to certain symptom characteristics such as: symptom nature, symptom severity, impact, and temporal aspects.
Psychological perspective	• Focus is on factors affecting the interpretation of sensations such as: internal frame of reference, attention to sensations, illness perception and susceptibility to suggestion.
Anthropological perspective	• Anthropologically situated research suggests that experiences and culture are in a continuous feedback relationship, where historical, political and/or social context contributes to different expectations and experiences. This influences when and how sensations are understood and acted upon as symptoms of disease. Moreover, it suggests that social and cultural structures may cause symptoms to manifest.
**Taking a broader view**	
General issues	• Symptom experiences are embedded within a complex interplay of biological, psychological and cultural factors.
	• Symptom interpretation in general practice is preceded by particular biomedical conceptualisations.
	• Research in symptoms as part of classifiable diseases cannot stand alone; symptom research should include symptoms as a generic phenomenon.
Interpretation of prevalence	• Surveys of symptom prevalence in the general population or in primary care reflect a variety of interpretations of sensations, which are not equivalent to expressions of underlying disease. We should be cautious and explicit when interpreting the findings.
Diagnoses	• If diagnosis of disease is based on the presence of specific symptom characteristics only, we may reinforce a dualistic approach (including medicalisation of normal phenomena and devaluation of medically unexplained symptoms).

#### ***Understanding symptom studies conducted in the general population***

The complex nature of how bodily sensations are assigned meaning as symptoms should be further integrated into the knowledge produced in symptom studies. We should be careful to treat individual subjective symptom presentations as objective signs of medical phenomena. For example some surveys make use of standardised symptom questionnaires asking only about the existence and duration of symptoms. Such observations may reflect a variety of interpretations by the respondents and need not be expressions of impact or illness. Hence, the observations may not be valid in a biomedical sense referring to a negative effect on health. Overall, the problem of validity is a serious, generic problem in assessments of individuals’ complaints as signs of disease [48,49]. In epidemiological terms, one could argue that the 'baseline condition’ framing bodily sensations and their potential transformation into symptoms is not the same for all individuals - be it patients or doctors. In anthropological terms, one would say that peoples’ interpretations of bodily sensations as symptoms are embedded within a particular social and cultural setting. Hence what we are actually measuring are differences in response to sensations more than an amount of signs of disease. We should, therefore be careful not to make simple interpretations of the 'symptom iceberg’ as a mass of unreported disease signs in the general population.

#### ***Symptom interpretation in general practice***

Similarly to studies of the general population, we may understand prevalence studies conducted in primary care better if we broaden our perspective. For example surveys of somatoform disorders in primary care waiting room populations report frequencies of 25-30% [50,51] whereas GPs report a prevalence about 15% [52,53]. This is often interpreted in the way that GPs overlook disorders, but we need to consider the possibility that symptom reporting by patients and doctors respectively, is not so much a presentation of the mass of reported disease signs as it is a difference in perspective on 'what counts as symptoms’. A reported 10-fold variation in GPs’ evaluation of symptoms as being medically explained or unexplained [53] may only be a demonstration of the fact that there is a gap between experience and biology, which is filled by social expectation, cultural categories and personal response [36,48].

Moreover, GPs often interpret symptoms in the context of consequences and it is a main task for primary care to identify serious disease as quickly as possible because delay in diagnosis may affect prognosis [54]. However, most patients seen in primary care present with symptoms without having any identifiable disease [55,56]. A biomedical approach to the interpretation of such symptoms may reinforce illness behaviour [57,58] and introduce risk of iatrogenic harm due to unnecessary tests and treatment [59,60]. Therefore, we need to improve the clinician’s ability to characterize symptoms according to outcome and actions needed. As presented in this paper, biomedical attempts to do this have been through the descriptions of “objective” symptom characteristics. On its own, this may be an insufficient way of capturing disease. Moreover, as described, psychological as well as socio-cultural factors may both cause the manifestation of symptoms, as well as amplify them. As patients experience and interpret bodily sensations outside the clinical encounter, it raises the question about how we on the one hand can decrease patient delay in serious diseases and how we on the other hand can improve treatment and avoid iatrogenic harm of patients with symptoms not fitting into well-defined disease categories.

#### ***Consequences of different perspectives for diagnoses***

The broadening of our understanding of symptoms also has consequences for diagnostic classification. Many diagnoses rely on the appearance and number of certain symptoms, e.g. Ménière’s disease and arthritis. The question is, whether symptoms make the basis of these diagnoses or the diagnostic classification just as much stimulate certain symptoms? It has been demonstrated repeatedly that functional somatic syndromes refer to the same underlying phenomenon [61,62]. Yet, the classification systems hold numerous syndrome diagnoses, each with their specific symptom pattern, e.g. irritable bowel syndrome, chronic fatigue syndrome and fibromyalgia. Patients seem to emphasize symptoms fitting with a diagnosed syndrome although they also present other symptoms when inquired about them [62,63]. While the clinician sometimes has to take a dualistic approach to symptoms and assess how they fit or do not fit with specific diagnostic categories, maybe primary care research should give higher priority to exploring symptoms as a phenomenon in its own right rather than focus on symptoms as part of diagnostic constructs only?

## Summary

As stated by Kroenke ’symptoms research is a fertile field’, but we need to be better aware and more explicit about how we understand symptoms. Physical characteristics of bodily sensations, as described in the biomedical sciences, are not sufficient basis for an interpretation; this applies both when the interpretation is made by the individual and by the GP. Psychological factors, context and cultural aspects also influence the interpretation of bodily sensations as symptoms and affect the related actions. These aspects must be taken into consideration when studies of symptom prevalence are conducted and evaluated. Research into 'symptom icebergs’ may further enlighten us on problems relating to the general practice setting, but we need a more in depth understanding of what we precisely mean when we talk about symptoms.

In relation to primary care, symptoms must be studied as a generic phenomenon. Symptom interpretation in general practice is embedded in biomedical conceptualisations. Much emphasis is put on symptom characteristics, but we must broaden our approach, both when making clinical assessments and diagnoses and when conducting symptom studies.

## Abbreviations

GP: General practitioner; SSAS: Somatosensory amplification scale.

## Competing interests

The authors declare that they have no competing interests.

## Authors’ contributions

All authors contributed to the idea, discussions and writing of the paper, MR and DJ primarily on biomedical issues, AFP on psychological issues and RSA on anthropological issues. All authors have read and accepted the final version of this article.

## Authors’ information

Marianne Rosendal is a GP, PhD and senior researcher in studies of medically unexplained symptoms and classification in primary care.

Dorte Jarbol is a GP, PhD and senior researcher in studies of symptoms and health care seeking behaviour with special focus on cancer alarm symptoms, irritable bowel syndrome and dyspepsia in population and primary care studies.

Anette Fischer Pedersen is a psychologist, PhD and postdoc in studies of healthcare seeking and the doctor-patient relationship.

Rikke Sand Andersen is an anthropologist, PhD and postdoc in studies of healthcare seeking and symptom experiences.

## Pre-publication history

The pre-publication history for this paper can be accessed here:

http://www.biomedcentral.com/1471-2296/14/167/prepub
